# Typhoid Fever and Its Prevention

**Published:** 1898-01-22

**Authors:** 


### Typhoid Fever and Its Prevention.

The recent discussion at the Royal Medical and Chirurgical Society on the prevention of enteric fever, although very interesting in many ways, was far too discursive to be taken as a complete presentment of the subject.

We were told rightly enough that typhoid excreta should be burned, or boiled, or in some efficient manner disinfected; that subsoils must not be fouled; that streams must not be polluted, and all the rest of it; but how, with our fouled streams, our filthy subsoils, and our undisinfected excreta, the present generation is to get water fit to drink we were not told at all, nor did the discussion touch the question of the efficiency and comparative utility of the means now in use for the manufacture of a potable water from sources which have already been exposed to chances of infection, viz., (1) dilution—the mixing of the water from each source with large quantities of water from other sources, a process which, although fairly influential as against epidemics, has but little effect in preventing the general prevalence of the disease; (2) sedimentation—which may be grouped along with dilution and delay in delivery under the head of storage; (3) filtration. These are the measures in which urban populations have to place their trust, and of these we heard practically nothing.

The three most interesting points which came out in the discussion related to the prolonged vitality of the germ of the disease, and its presence in apparently healthy people; to the possible agency of flies in causing the high typhoid rate in the so-called "midden towns"; and to the grave doubts thrown upon the safety of a proceeding which has for long received the sanction of leading sanitary authorities, namely, the abolition of cisterns and the substitution of the plan of drawing water direct from the main. The latter is a point of great importance, because there is much reason to fear that in the attempt which has been made to get rid of the undoubted evils connected with dirty cisterns, we have exposed our general water supplies to considerable risk of serious pollution.

Again and again it has been said, and with perfect truth, that dirty cisterns are the cause of much unhealthiness, and cisterns whose overflows are directly connected with drains have been repeatedly shown to be sources of actual disease. One of the main grounds, indeed, upon which the "constant" system of water supply has been advocated by many sanitarians, has been that by it alone is it possible to get rid of the cistern with all its nastiness.

Where there are no cisterns, all taps are of course connected directly with the main, but even where cisterns have been retained, the plan which has received official sanction has been the fixing of a "draw tap on the supply pipe," by means of which "water can always be obtained for drinking and cooking purposes in a pure and cool condition direct from the main." (Report to the Local Government Board.)

What was pointed out, however, in the discussion was that the large extension of this plan has introduced a new and very serious danger of polluting the whole mass of water in the mains themselves.

However pure the water of a public supply may be when it arrives at its point of distribution, it may be, and it often is, most grievously befouled by bad arrangements of the taps and fittings. Even the most "constant" water supply must be interrupted now and again, and when this happens all sorts of filth, from all sorts of open taps, is apt to be drawn up into the mains. If the public supply always ended with a cistern and a ball tap, at least nothing could be sucked back when the supply was cut off. But taps are now placed on the main, and these, however useful to the householder when the water is "on," are a danger to the community when it is cut off, for then whatever filth the tap opens into will be sucked up into the pipe, for general distribution afterwards. Instances of this sort might easily be given, instances which, however, are far too unpleasant to detail. One only need be mentioned as illustrating how real this danger is. It was reported that at times the tap on the rising main of a certain house supplied blood instead of water! Yet this was true, for on investigation it was found that when the water was turned off the pipes sucked up the blood from a neighbouring slaughter-house, and distributed it to the surrounding houses; and, in fact, one has but to think to see chances of local pollution of our water supplies on every side. This is a matter in regard to which no householder can protect himself, yet it is one in regard to which all householders may fairly ask to be protected.

